# Efficacy of Arterial Embolization prior to Pancreaticoduodenectomy for Pancreatic Arteriovenous Malformation: A Case Report

**DOI:** 10.70352/scrj.cr.24-0117

**Published:** 2025-03-18

**Authors:** Ryota Kiuchi, Takanori Sakaguchi, Toshiki Kawabata, Osamu Jindo, Akihiro Uno, Atsuko Fukazawa, Keigo Matsumoto, Junichi Kaneko, Daijiro Suzuki, Yoshihisa Ookawa, Kenshi Kawamura, Shioto Suzuki, Shohachi Suzuki

**Affiliations:** 1 Department of Gastroenterological Surgery, Iwata City Hospital, Iwata, Shizuoka, Japan; 2 Department of Gastroenterology, Iwata City Hospital, Iwata, Shizuoka, Japan; 3 Department of Diagnostic Radiology, Iwata City Hospital, Iwata, Shizuoka, Japan; 4 Department of Radiology, Hamamatsu University School of Medicine, Hamamatsu, Shizuoka, Japan; 5 Division of Pathology, Iwata City Hospital, Iwata, Shizuoka, Japan

**Keywords:** pancreatic arteriovenous malformation, arterial embolization, pancreaticodudenectomy, *Schistosomiasis japonica*

## Abstract

**INTRODUCTION:**

Pancreatic arteriovenous malformation is a rare disease characterized by abnormal vascular connections between arteries and veins. Despite the risk of increased intraoperative blood loss due to abundant blood flow, surgical resection remains the only curative modality for pancreatic arteriovenous malformation. We present a case of pancreatic arteriovenous malformation in which subtotal stomach-preserving pancreaticoduodenectomy was successfully performed following selective arterial embolization to reduce intraoperative blood loss.

**CASE PRESENTATION:**

A 53-year-old Southeast Asian man was referred to our hospital with abdominal pain. Contrast-enhanced computed tomography revealed enhancement of the pancreatic head and superior mesenteric vein in the early arterial phase, suggesting the presence of an arteriovenous malformation. Maximum intensity projection images and angiography revealed arterial branches from the gastroduodenal artery and superior mesenteric artery to the arteriovenous malformation, subsequently draining into the portal venous circulation. We supposed that abdominal pain resistant to medical treatment was due to the pancreatic arteriovenous malformation, and surgical resection was deemed necessary. Subtotal stomach-preserving pancreaticoduodenectomy was safely performed on the day after arterial embolization of pancreatic arterial branches to reduce intraoperative blood loss. The procedure resulted in an intraoperative blood loss of 336g. The patient was discharged on the 16th postoperative day with no complications and has not experienced abdominal pain since.

**CONCLUSIONS:**

Selective arterial embolization prior to pancreaticoduodenectomy against pancreatic arteriovenous malformation is a safe and feasible procedure to reduce intraoperative blood loss.

## Abbreviations

AIPDA
: anterior inferior pancreaticoduodenal artery
ASPDA
: anterior superior pancreaticoduodenal artery
AVM
: arteriovenous malformation
CT
: computed tomography
GDA
: gastroduodenal artery
MIP
: maximum intensity projection
PIPDA
: posterior inferior pancreaticoduodenal artery
PSPDA
: posterior superior pancreaticoduodenal artery
SMA
: superior mesenteric artery
SMV
: superior mesenteric vein
SSPPD
: subtotal stomach preserving pancreaticoduodenectomy

## INTRODUCTION

Arteriovenous malformation (AVM) is a rare disease characterized by abnormal vascular connections between feeding arteries and veins. AVMs of the gastrointestinal system are most frequently observed in the cecum and right colon. In contrast, pancreatic AVMs, first reported by Halpern et al. in 1968,^1)^ are relatively uncommon. AVM causes abdominal pain, upper gastrointestinal ulcers, gastrointestinal bleeding, and pancreatitis. Surgical resection is often necessary in patients resistant to medical treatment. However, the risk of excessive intraoperative blood loss due to abundant arterial blood flow into pancreatic AVMs should be considered. To mitigate this risk, it is important to reduce the abundant arterial blood flow by arterial embolization prior to surgical resection.

Herein, we present a case of pancreatic head AVM that was successfully treated with selective arterial embolization followed by subtotal stomach-preserving pancreaticoduodenectomy (SSPPD).

## CASE PRESENTATION

**Fig. 1:**
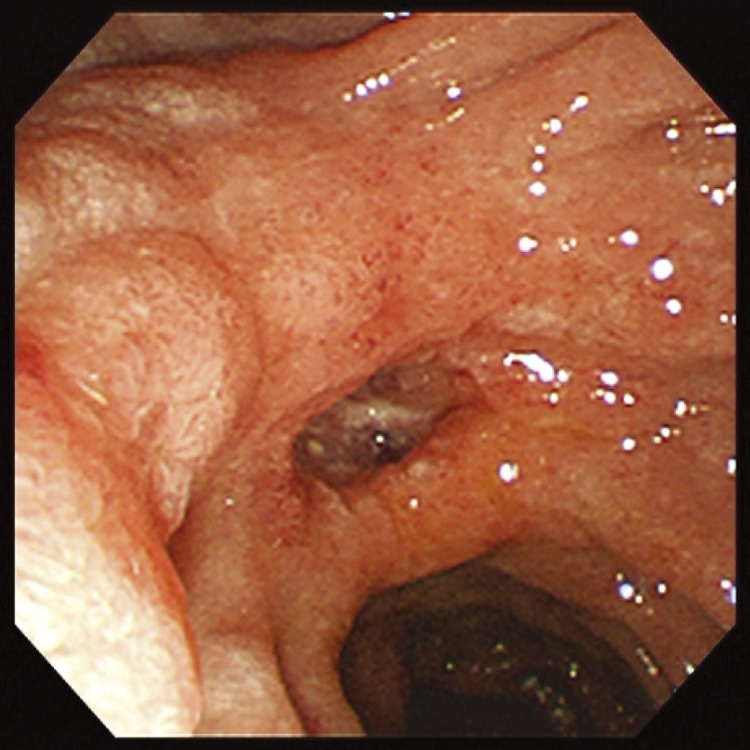
Findings of upper gastrointestinal endoscopy. Upper gastrointestinal endoscopy revealed a large ulcer at the duodenal bulb.

**Fig. 2:**
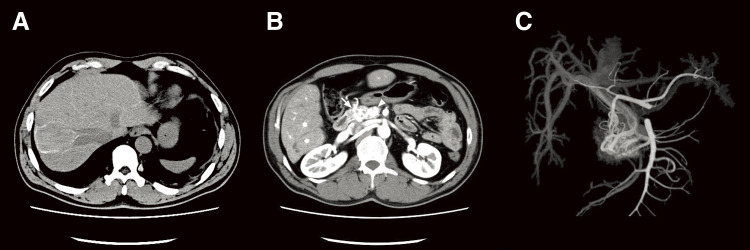
Findings of computed tomography (CT). (**A**) Non-enhanced CT showed the presence of intrahepatic reticulated calcification, indicating the presence of *Schistosomiasis japonica*. (**B**) Contrast-enhanced CT demonstrated the enhancement of the pancreatic head (arrow) and main portal vein (arrowhead) in the late arterial phase. (**C**) Maximum intensity projection images revealed blood flow from the gastroduodenal artery and superior mesenteric artery to the main portal vein.

**Fig. 3:**
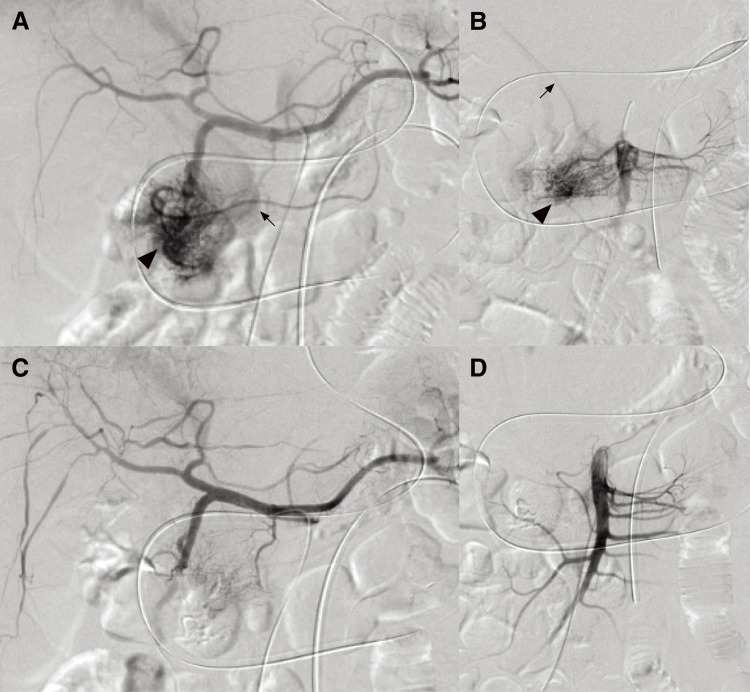
Findings of angiography. (**A**) Angiography of the celiac axis showed a markedly proliferative vascular network at the pancreatic head (arrowhead) via the gastroduodenal artery and early drainage into the main portal vein (arrow), suggesting the existence of an AVM. (**B**) Angiography of the SMA also showed AVM (arrowhead) via the AIPDA and PIPDA, as well as early drainage into the main portal vein (arrow). (**C** and **D**) After embolization using gelatin sponges, AVM was not visible on angiography of the celiac axis (**C**) or SMA (**D**). AIPDA, anterior inferior pancreaticoduodenal artery; AVM, arteriovenous malformation; PIPDA, posterior inferior pancreaticoduodenal artery; SMA, superior mesenteric artery

**Fig. 4:**
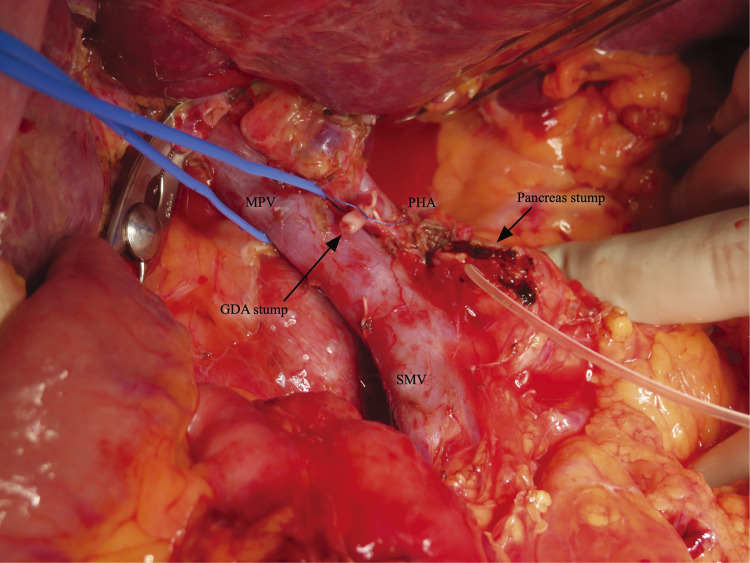
Intraoperative findings. The intraoperative findings did not demonstrate the impact of arterial embolization on the pancreatic parenchyma. GDA, gastroduodenal artery; MPV, main portal vein; PHA, proper hepatic artery; SMV, superior mesenteric vein

A 53-year-old Southeast Asian man presented to a local physician with abdominal pain. He had no specific previous medical history or prior treatments. Abdominal ultrasonography revealed hepatomegaly. Since his abdominal pain was resistant to any medication, he was referred to our hospital for a comprehensive examination and subsequent treatment. Upper gastrointestinal endoscopy revealed an ulcer in the duodenal bulb (**Fig. 1**), although he had been taking a histamine H2 antagonist. *Helicobacter pylori* infection was not detected in any examinations. Plain abdominal computed tomography (CT) revealed intrahepatic reticulated calcification (**Fig. 2A**), indicating the presence of *Schistosomiasis japonica*. Contrast-enhanced CT demonstrated early enhancement of the pancreatic head and superior mesenteric vein in the late arterial phase (**Fig. 2B**). Maximum intensity projection (MIP) images revealed blood flow from the gastroduodenal artery (GDA) and superior mesenteric artery (SMA) to tortuous vessels, subsequently draining into the portal venous circulation (**Fig. 2C**). Based on these results, we diagnosed the patient with pancreatic AVM. His abdominal pain persisted despite treatment with oral acetaminophen and proton pump inhibitors. Continuous intravenous fentanyl injection relieved his abdominal pain. However, the duodenal ulcer did not improve upon the second upper gastrointestinal endoscopic examination. We considered that the duodenal ulcer was likely caused by the abnormal peri-pancreatic circulation due to the pancreatic AVM, and surgical resection of the pancreatic head was needed to alleviate his symptoms. Considering the risk of increased intraoperative blood loss due to abundant arterial blood flow caused by the presence of the AVM in the pancreatic head, preoperative arterial embolization was scheduled for the day before surgery. Angiography revealed the existence of the AVM in the pancreatic head, which was supplied by the GDA and anterior/posterior pancreaticoduodenal artery (AIPDA/PIPDA) branching from the SMA (**Figs. 3A** and **3B**). After selective arterial embolization of the GDA, APIDA, and PIPDA using gelatin sponges (Serescue; Nippon Kayaku, Tokyo, Japan), AVM was no longer visible under angiography of the celiac axis or SMA (**Figs. 3C** and **3D**). We did not use coils because we were concerned that intra-arterial coils might hamper arterial ligation and division. No complications occurred after arterial embolization until the next day. Since there were no abnormal laboratory data just before surgery, SSPPD was performed. After laparotomy, GDA and peripancreatic arterial branches from SMA were firstly divided to reduce the pancreatic blood flow. The intraoperative findings did not demonstrate any impact of arterial embolization on the pancreatic parenchyma (**Fig. 4**). The operation time was 453 min, and the blood loss was 336g. The patient was discharged on the 16th postoperative day with no complications and has not experienced abdominal pain since.

**Fig. 5:**
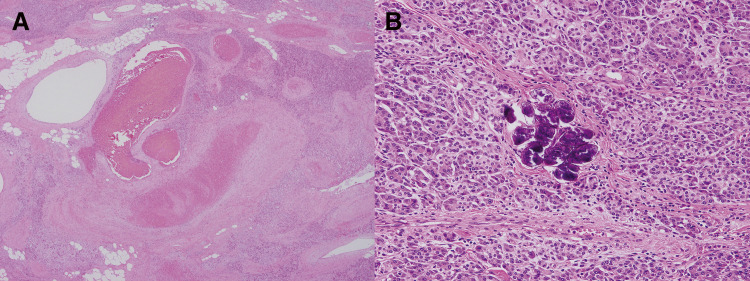
Pathological findings of resected specimens (hematoxylin and eosin staining). (**A**) Dilated vessels of unequal size were found in the pancreatic parenchyma, consistent with the finding of arteriovenous malformation. (**B**) Eggs of *Schistosoma japonicum* were observed in the resected pancreatic tissue.

Pathological examination revealed the presence of dilated vessels of unequal sizes, which was consistent with the features of pancreatic AVM (**Fig. 5A**). Eggs of *Schistosoma japonicum* were observed within pancreatic tissue and in the dissected lymph node around the pancreatic head (**Fig. 5B**).

## DISCUSSION

AVM is a rare disease characterized by abnormal vascular connections between arteries and veins. Pancreatic AVM was first reported by Halpern et al. in 1968.^1)^ Previous reports showed that pancreatic AVMs mainly occur in middle-aged Asian males. Although pancreatic AVMs occurred throughout the pancreatic parenchyma, 59.4% and 33.3% of pancreatic AVMs were found in the head and body/tail, respectively. In the remaining 7.2% of cases, pancreatic AVMs occupied the entire pancreatic parenchyma from head to tail.^2,3)^ The primary cause of pancreatic AVMs is congenital origin (90.5%), including Osler–Weber–Rendu disease. Congenital AVMs are believed to be caused by abnormal formation of arteriovenous plexuses during embryonic development.^2)^ The remaining 9.5% of this disease is thought to be acquired and caused by pancreatitis, trauma, and tumors.^4)^ In this case, radiological and pathological examinations revealed the presence of *Schistosoma japonicum* in the hepatic and pancreatic parenchyma and peri-pancreatic lymph nodes. Vascular remodeling in the pancreatic parenchyma may be derived by the signaling cascade via several chemical mediators, such as interleukin-13 and transforming growth factor-β, triggered by *Schistosoma* antigen.^5,6)^ Based on these findings, *Schistosomiasis japonica* may be a causal factor for the development of pancreatic AVMs.

Several radiological examinations have been performed to diagnose pancreatic AVMs. Recently, due to advancements in CT (time and spatial resolution) and image-analyzing software, contrast-enhanced multi-detector CT has become sufficient for diagnosing pancreatic AVMs.^7)^ Three-dimensional analysis of contrast-enhanced multi-detector row CT images easily identifies the input arteries and the drainage vessels. Since we had already identified the AVM-associated vessels using enhanced multi-detector row CT images and computer-analyzed images, especially in MIP images,^8)^ interventional angiography was easily performed.

Surgical resection and arterial embolization have been reported as treatment modalities for pancreatic AVMs. Although arterial embolization is a minimally invasive procedure, it is difficult to embolize all AVM-associated arteries. When conservative treatment and arterial embolization are ineffective, surgical resection should be considered. However, excessive intraoperative blood loss due to abundant peri-pancreatic arterial flow is a serious hazard. In the literature, 2 studies reported that intraoperative blood loss during pancreaticoduodenectomy for AVM in the pancreatic head without arterial embolization was 1260 and 1950g, respectively.^9,10)^ Additionally, 2 reports described the effectiveness of preoperative arterial embolization in reducing the intraoperative blood loss during resection of AVM in the pancreatic head. Intraoperative blood loss during pancreaticoduodenectomy for AVM in the pancreatic head with preoperative arterial embolization of the GDA alone was 605g in 1 report,^11)^ and another report did not describe the blood loss.^12)^ In contrast to the 2 reports, we almost completely embolized AVM-related arteries via GDA, AIPDA, and PIPDA. After complete embolization, we considered the risks of arterial embolization, such as pancreatic necrosis or pancreatitis due to arterial ischemia.^13)^ To avoid such risks associated with ischemia, we scheduled surgery for the day after arterial embolization. Fortunately, our patient did not show any complications related with arterial embolization until the next morning following embolization. As a result, SSPPD was safely performed, and the intraoperative blood loss was 336g. Thus, we strongly believe that the preoperative arterial embolization is safe and effective in reducing the intraoperative blood loss during resection of pancreatic AVMs. Moreover, we addressed the responsible arteries first to minimize the risk of massive bleeding as much as possible because the artery-first approach could reduce the intraoperative blood loss.^14,15)^ Based on these points, we recommend planned arterial embolization before resection of pancreatic AVMs.

## CONCLUSIONS

In this case, selective arterial embolization before SSPPD for pancreatic head AVM was performed to reduce intraoperative blood loss. No complications were observed after arterial embolization or surgery. Selective arterial embolization prior to pancreaticoduodenectomy for pancreatic AVMs is a safe and feasible procedure to reduce intraoperative blood loss.

## DECLARATIONS

### Funding

This study did not receive any funding.

### Authors’ contributions

RK was involved in the clinical practice, conception, design, and acquisition of data.

TS, JK, DS, YO, KK, and ShiotoS were involved in the clinical practice and conception and design.

TK, OJ, AU, AF, KM, and ShohachiS approved the final version of the manuscript.

All authors have read and approved the manuscript, and they are responsible for the manuscript.

### Availability of data and materials

The datasets used in this study are available from the corresponding author upon reasonable request.

### Ethics approval and consent to participate

This work does not require ethical considerations or approval.

### Consent for publication

Informed consent was obtained from the patient for publication of this case report.

### Competing interests

The authors declare that they have no competing interests.
